# 

**Published:** 1853-09

**Authors:** 


☞ Postmasters are requested to return the numbers sent to those, who do not consider themselves subscribers.

